# The efficacy of Mohs micrographic surgery over the traditional wide local excision surgery in the cure of dermatofibrosarcoma protuberans

**DOI:** 10.11604/pamj.2019.33.297.17692

**Published:** 2019-08-13

**Authors:** Malumani Malan, Wu Xuejingzi, Song Ji Quan

**Affiliations:** 1 Department of Dermatology and Venereology at Zhongnan Hospital of Wuhan University, Wuhan City, Hubei Province, Peoples Republic of China; 2 Livingstone Central Hospital, Southern Province, Zambia; 3 Department of Dermatology and Venereology at Zhongnan Hospital of Wuhan University, Wuhan City, Hubei Province, Peoples Republic of China; 4 Head of Department of Dermatology and Venereology at Zhongnan Hospital of Wuhan University, Wuhan City, Hubei Province, People’s Republic of China

**Keywords:** Dermatofibrosarcoma, dermatofibrosarcoma protuberans (DFSP), Darier-Ferrand, wide local excision (WLE), Mohs micrographic surgery (MMS), recurrence rate

## Abstract

Usually most patients with dermatofibrosarcoma protuberans (DFSP) may present rather late when the tumor is in protuberant phase due to its rarity and indolent onset. It has a high propensity for local recurrence and destructive nature. Management of DFSP requires a biopsychosocial and Multidisplinary approach regardless of the clinical or immunohistochemical variant. Surgery is the Gold standard management of localized disease. DFSP rarely exhibits any lymphatic or hematogenous dissemination. It is because of its high recurrence rate associated with Wide Local Excision (WLE), the introduction of Mohs micrographic surgery (MMS) has really helped in reducing the rates of recurrence of DFSP. Thus, the aim of this meta-analysis and systemic review is to advocate for MMS over WLE for DFSP and other cutaneous malignancies using DFSP as a prototype. The objective of this study were to conduct a meta-analysis on comparative surgical methods used in the cure of DFSP with regards to WLE verses MMS, to evaluate the cure rates with relation to recurrence rates, offer a recommendation on the various treatment modalities based on the location of lesion, and use of adjuvant therapy in different clinical-medical setups. A comprehensive retrospective analysis search in EMBASE, Google Scholar and Medline (PubMed) for studies published from 2008 to 2018 containing the surgical management of DFSP with WLE verses MMS were reviewed. Five studies of moderate-quality evidence (level B) with a pooled patient load of 684 was analyzed and found for recurrence of DFSP after WLE and MMS to be 9.10% and 2.72% respectively after an average follow-up time for both groups of 5.32 years with a female predominance of 1.58. The trunk is the commonest site for the DFSP lesion which was at 52.80% then the upper and lower extremities zones and the head and neck zones at 31.75% and 15.45% respectively. The pooled adjusted odds ratio (OR) analysis indicated that there was a direct relationship with regards the reduced recurrence rate of DFSP in the MMS group compared to the WLE group (OR:0.31;95%; CI :0.17-0.56). Furthermore, there was significant association between the reduced recurrence rate with the MMS in DFSP patients with a statistical P-value of 0.0001 at 95% CI. The expected increased recurrence rate by zones was in WLE head and neck zone at 38.19% then trunk and extremities zone at 13.34%. In the MMS group it was at of 23.4% as compared to 16.0% in the head and neck zone. Mohs Micrographic Surgery (MMS) is more efficacious in the cure rate and recurrence reduction of DFSP and should be advocated for as first line therapy especially in high recurrence prone zones.

## Introduction

Dermatofibrosarcoma protuberans (DFSP) is one of the rare, indolent, spindle cell mesenchymal sarcoma or malignancy of low-grade aggressiveness, that arises in the dermis and can extend to the deep subcutaneous tissue and can also affect other under lying structures like muscles and bones in un intervened cases and often stains positive for CD34 [1-3]. It was first described by Darier and Ferrand however Hoffman officially coined the term dermatofibrosarcoma protuberans [4]. DFSP, described as a slow-growing low-grade cutaneous sarcoma [5] that often presents or has a predilection on the following sites: trunk and proximal extremities [6], less frequently on the head and neck regions [2,7,8]. DFSP has a very low potential to metastasize but with significant subclinical tentacular extensions and great capacity for local destruction as evidenced by Acosta *et al.* [9]. Usually patients may present rather late when the tumor is already several centimeters in size due to its indolent onset. Misdiagnoses of the tumor for a simple scar, keloid [10], or cyst, lump is often the case [11]. As it has been shown to arise from regions or areas of previous trauma. Even when allowed to grow for many years, the tumor usually remains asymptomatic. Cytogenetically, these tumors have been associated with chromosomal translocation at position 17:22 leading to an overexpression of tyrosine kinase PDGFB, which can be targeted with Imatinib, a tyrosine kinase inhibitor [12].

This rare cutaneous tumor, constitutes of not more than 0.1% of all malignancies and approximately 1% of all soft-tissue sarcomatous tumors [8,13]. It is a locally aggressive sarcoma of intermediate malignancy that favors young to middle-aged individuals of all sexes [14]. There has been reports of lesions that are present at birth or with an early onset during childhood [15,16]. Loghdey MS *et al.* describes DFSP as the most common skin tumor with prevalence of about 0.8 to 4.2 cases per million persons per year and it roughly accounts for between 2 and 6% of all soft tissue sarcomas [17]. The incidence of DFSP ranges between 0.5 and 1:100,000, thus it is considered as the most common cutaneous sarcoma. There seems not to be a gender or racial predilection for the tumor. However, the pigmented variant (referred to as Bednar's tumor) is more common in black population [18]. The hypothesized pathogenesis of DFSP is largely due to protooncogenes which are as a result or is associated by either; marked by chromosomal translocation t (17;22) (q11; q13.1) or an extra ring of chromosome derived from the t (17;22) which ends in the formation of COL1A1-PDGFB fusion gene in the greater number of cases of DFSP [19-21]. The chromosomal translocation is found in more than 90% of cases, and involves 17q22 and 22q13 [3,22]. The chimeric protein COL1A1-PDGFB, is processed into a functional beta platelet-derived growth factor (PDGFB) ligand that in turn causes PDGFRB signaling activation through an autocrine stimulation loop in tumor cells as evidenced by Simon *et al.* [23-25]. The COL1A1-PDGFB fusion gene, exhibit growth factor activity, this furthers on the proliferation on tumor cells [26]. Molecular studies have shown that fusion genes are crucial as the initiating factors of tumorigenesis in many translocation-related sarcoma subtypes [24,27]. Once PDGFB is cleaved from the COL1A1-PDGFB chimeric protein, it stimulates tumor cells to go into an autocrine fashion, thus, leading to proliferation transformation [28]. There are some documented risk factors for developing DFSP, some cases develop at previous trauma site and reports have included a burn scar and the vaccination site [29], central venous lines sites [30]. Exceptional cases have been associated with previous radiotherapy to the area. There is an association between DFSP and children with adenosine deaminase deficient severe combined immunodeficiency. Patients affected by the latter have a higher incidence of tumors presenting at early age and often multicentric [31].

The clinical presentation is somewhat uniform with a few variations in racial and individual genetic variations. The tumor is more often commonly affects the trunk in about 40%-50% of cases, the proximal extremities (30%-40%), and the least being head and neck at 10%-15% of cases [32,33]. Involvement of the limbs is usually on the proximal aspects. Presentation on the hands and feet, particularly on the digits, is very rare. Clinically, classical DFSP tumors start as a plaque, which sometimes may be atrophic in nature. It may begin in early adulthood as one or more small, firm, painless, flesh-colored or erythematous dermal nodules [34]. Their progression is most of the time very slow and may occur over many months to years; a significant proportion of these tumors only become protuberant after a long period of time. Jonathan *et al.* classified DFSP into three different forms: the initial phase as morphea-like form resembling a scar, morphea, morphea-form basal cell carcinoma. Secondly, dermatofibroma plaque; this is an atrophoderma-like form similar to atrophoderma or an anetoderma. Finally, an angioma-like form resembling vascular malformations [35]. Subsequently, one or multiple nodules may mushroom in the protuberant phase. These nodules grow, extend and coalesce, becoming more redder or bluish as they enlarge to form an irregular protuberant swelling. At this phase, the base of the tumor is a hard-indurated plaque of irregular outline. In advanced stages, a proportion of some lesions become painful and may be associated rapid growth, ulceration and exudation [36]. Typically, DFSP lesions ranges between 1 to about 6 cm in size. However, in some occasions, and if not remedied earlier, these tumors may grow to as much as 20 cm in diameter with multiple satellite nodules. The overlying skin is fixed to the tumor, but not to deeper structures. However, it has been documented that long-standing or recurrent tumors may invade deeper structures like fascia, muscle, periosteum, and even bone tissues [37,38]. On physical exam, initially the tumor is freely moveable from the underlying surface. As the tumor subsequently evolves in size, it becomes adherent to the underlying surface. At this stage the overlying epidermis may be thinned and telangiectasias appear. Bleeding and ulceration are uncommon. DFSP may less frequently present like a non-protuberant, violaceous and atrophic lesion similar to a sclerosing like basal cell carcinoma or morphea; which is a common presentation in childhood. The pigmented variant of DFSP is termed Bednar tumor.

Diagnosis of DFSP can be achieved according to clinical, histopathological and immunohistochemical findings in any case variant of DFSP. After a tentative diagnosis of DFSP, a comprehensive evaluation of the patient is crucial for an effective management of the patient. Rarely does DFSP exhibits any hematogenous or lymphatic dissemination as observed by Gloster *et al.* [39,40]. Prior any surgical procedure, magnetic resonance imaging (MRI) is important, which is extremely sensitive than physical examination, for ascertaining tumor involvement to surrounding tissues and structures especially in evaluation of lesions in locations like the head and neck regions and some upper part of the trunk [41,42]. However, Computed tomography (CT) is usually preferred in rare cases of suspected bone involvement. Fluorescence in situ hybridization (FISH) and reverse transcriptase polymerase chain reaction (RTPCR) are reserved as screening tools for the presence of COL1A1-PDGFB fusion gene prior to initiation of oral imatinib in molecular targeted therapy. For lesions devoid of the classic t (17,22) translocation mutation, they respond poorly to imatinib therapy [4,43]. Llombart *et al.* alludes that the diagnostic criteria of DFSP is usually established on the account of positive histopathological and immunohistochemical findings. Positive immunohistochemical expression of CD34 is considered characteristic for the diagnosis of DFSP. It is approximated that over 80% to 100% of DFSP express this marker, although between 10% and 20% are negative, as mostly noted in the fibrosarcomatous type [44,45]. Nonetheless,CD34 expression has been increasingly reported in other non DFSP sarcomas [46,47], such as myofibrosarcoma [48], nuchal-type fibroma [49], inflammatory myofibroblastic tumor, epithelioid, or angiosarcoma. Positive CD34 has been reported in some benign fibrohistiocytic lesions, like in the solitary fibrous tumor, sclerotic fibroma, cellular digital fibromas [50], superficial acral fibromyxomas [47], and dermatofibromas [51]. As such, this marker may soon be considered less specific for DFSP but a careful clinical and immunohistochemical evaluation is thus essential in the definitive diagnosis of DFSP. In the management of DFSP a biopsychosocial and Multidisplinary approach regardless of the clinical or immunohistochemical variant is required. Surgery has always been the mainstay of management of localized disease. At first contact or attempt, complete surgical resection is advisable at all cost [52,53]. It is because of its high recurrence rate associated with the traditional wide local excision(WLE) , the introduction of Mohs micrographic surgery (MMS) has really helped in reducing the rates of recurrence of DFSP to as low as less than 1% and a range of 0% to 8.3% [4,39,54,55]. Thus, the aim of this meta-analysis and systemic review is to advocate for MMS over WLE for DFSP and other cutaneous malignancies using DFSP as a prototype.

## Methods

This Meta-analysis was done according to the preferred Reporting items for systematic reviews and meta-analyses known as the PRISMA statement [56].

**Study selection**: a comprehensive search in EMBASE, Google Scholar and Medline (PubMed) for studies and literature search from the 08^th^ June, 2018 to the 01^st^ September, 2018 was conducted. Articles in English or French (could be translated) published from 2008 to 2018 containing the search key words “dermatofibrosarcoma”, “dermatofibrosarcoma protuberans”, and “Darier-Ferrand” were sought. Surgical management of DFSP. The search field was under the following research types: Randomized Controlled Trials (RCT), clinical trials, comparative studies, Controlled Clinical Trials (CCT), Meta-analysis, observational studies, systematic reviews and reviews. It was further limited to publications indexed with the Medical Subject Heading terms or Mesh search headings used were: “recurrence”, “conventional surgery” or “Wide Local Excision (WLE) and Mohs micrographic surgery (MMS)”, “MMS vs WLE”. This systematic review and search were based on PubMed.

**Inclusion criteria**: eligibility to our study were any RCTs comparing a 10-year recurrence rates of WLE to MMS in patients having primary or secondary DFSP on any part of body. In an advent that inadequate number of RCTs were found, non-randomized trials (NRTs) were to be included as long as they compared recurrence rates associated with MMS vs WLE (comparative NRTs) or reported only recurrence rates associated with MMS (noncomparative NRTs). The study should include more than ten patients.

**Exclusion criteria**: any studies involving adjuvant therapy (Radiotherapy or chemotherapy), fewer than 10 patients, incomplete data outcomes or either poor methodological design, duplicate publications, review articles and those conducted over 10years ago were excluded. Also, case reviews, pre-clinical studies, titles, abstracts and expert opinions were not accepted in our meta-analysis.

**Data extraction and quality assessment**: the data were independently extracted by one reviewer (MM) from each of the selected studies using an Excel spreadsheet and the second (WXJ) and third reviewer (SJQ) verified them. The extracted data included general characteristics, recurrence rates, types of surgical methods used. Grey areas in the investigation concerning outcomes of interest were reviewed later and the consensus was reached by reverification by the investigator. In our study, MMS was described as sequential tumor removal, followed by meticulously histopathologic examination of the entire excisional tumor margins regardless of the examiner of the slides (histopathologists or MMS surgeon) or the excisional and histologic technique either cryostat or paraffin. While wide local excision (WLE) was described as the traditional excision of a clinical tumor with well predefined margins and histologic examination of the specimen using random vertical sections such as the quadrant method or bread-loafing technique. The quality assessment of diagnostic accuracy studies 2 (QUADAS-2) tool was employed to assess the quality of the included studies; where every study item was scored as: low risk (L), high risk (H) or uncertain risk (U).

**Statistical analysis**: the random effects model (Mantel-Haenszel Model) was adopted in this study because of difference in sample size, anti-angiogenic therapy and reference standards among the included studies. The analysis was done using review manager (version 5.3), Meta-Disc (version 1.4) software and Microsoft Excel 2016 (Microsoft, Seattle, WA, USA). Meta-analyses were performed using review manager (version 5.3), The Cochrane Collaboration, The Nordic Cochrane Centre, Copenhagen. Heterogeneity was estimated (I 2), and a random effects model was used if heterogeneity testing revealed significant results. A chi-square test was performed for categorical variables and Student's t-test was performed for continuous variable using [Review Manager (version 5.3) software and Microsoft Excel 2016 (Microsoft, Seattle, WA, USA].

To analyze the association of the reduced recurrence rate in MMS with increased chances of cure rate at 5 years, the adjusted odds ratio (OR) were used. The pooled OR [and 95% confidence interval (CI)] was estimated using a weighted random-effect model (the Mantel-Haenszel approach). Heterogeneity among the studies was assessed by Cochran Q and I2 statistics (I2 = (Q-df)/Q × 100%; I2 < 25%, no heterogeneity; I2 = 25-50%, moderate heterogeneity; I2 = 50-75%, large heterogeneity, I2 > 75%, extreme heterogeneity). The heterogeneity was considered significant if either the Q statistic had p < 0.1 or I 2 > 50%. Visual inspection of asymmetry in funnel plots was conducted to evaluate publication bias. The meta-analyses were conducted with the software Review Manager 5.2 (The Cochrane Collaboration, 2011) to evaluate the superiority of MMS over WLE in the management of DFSP.The recurrence rate was also used to establish the high cure rate of MMS by associating it with reduced rate of reoccurrence in cases that underwent MMS as opposed to those that opted for WLE for whatever reason. The quality of our studies was assessed with sensitivity and bias analysis. The risk of bias table of the included studies was independently assessed according to the Cochrane Handbook for Systematic Reviews of Interventions by two members, MM and WXJ. Also, if any occurrence of poor agreement and no consensus could be achieved, a third investigator was the adjudicator (SJQ).

**Figure 1:**
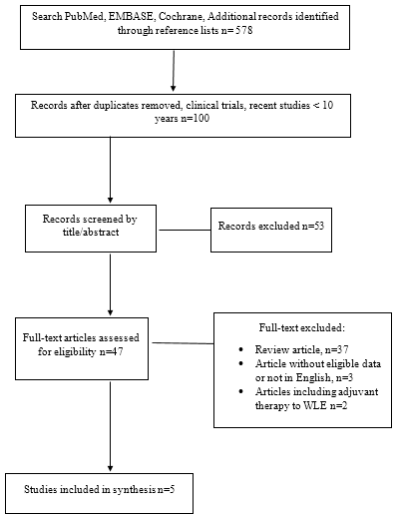
Flow diagram of the five retrospective comparative non randomised studies included in the meta-analysis

Figure 1 depicts a flow chart of selected studies according to the PRISMA criteria. Five hundred and Seventy-Eight studies were identified through the initial search strategy and reviewing reference lists, respectively, of which 478 were not eligible in view of our screening of titles, abstracts or because of duplicated records. Thus, we retrieved the full text of 100 studies. Of these, 53 articles were further excluded because they were older than 10 years. Further 37 articles were excluded because they were review articles as well as case studies and others had less than 10 subjects in their studies. Articles without eligible data or not in English were three. Articles including adjuvant therapy to WLE and also recurrence case were two. Consequently, five studies were eligible for this meta-analysis review. Due to the aforementioned reasons, none of the included studies were randomized controlled trial. All articles were retrospective comparative NCTs studies. The characteristics and patient demographic information from each of the five studies included in our meta-analysis are shown in Table 1 [57-59]. The eligible studies were articles published between 2008 and 2018, and had sample sizes range of 48 to 284.Our study showed a female predominance of 1.58 in a population study of 684 with an average age group of the patients being 44.6 years for both categories of wide local excision (WLE) and those of Mohs microscopic surgery (MMS). An average follow-up time for both groups was 5.32 years. The pooled recurrence rate for DFSP in the WLE was 9.10% and in MMS group was 2.72%. The study did not document the location of the lesions (Table 2). However, it was observed that none of the patients had significant difference after adjusting confounding factors in a multivariate logistic regression model (data not shown). In the meta-analysis, the pooled adjusted odds ratio (OR) analysis indicated that there was a direct relationship with regards the reduced recurrence rate of DFSP in the MMS group compared to the WLE group (OR: 0.31;95% CI: 0.17-0.56) as shown in Table 3 [57-59] (the forest plot). Furthermore, there was significant association between the reduced recurrence rate with the MMS in DFSP patients with a statistical P-value of 0.0001 at 95% CI. The studies included in our study went through a quality assessment of diagnostic accuracy of studies as shown in Table 4 [57-59].

**Table 1:** Characteristics features and recurrence rates of our meta-analysis, by type of surgery

Studies and Year	DuBay *D et al.*	Foroozan *et al.*	Meguerditchian *et al*.	Paradisi *et al*.	Veronese *et al*.
**Study Design**	Retrospective Review	Retrospective Study of NRCT	Retrospective Review	Retrospective Trial	Retrospective Review
**Country**	U.S. A	U.S. A	UK, Canada	Italy	Italy
**Number of Patients**	158	264	48	79	135
**Age group**	40 (3-88)	53 (29-73)	40	44 (10-83)	46 (7-86)
**Women to Men ratio**	2.24	2.05	1.18	1.26	1.18
Site of lesion					
**Trunk**	44.6%	NA	39.6%	63.3%	63.7%
**Extremities**	36.8%	NA	37.5%	25.3%	27.4%
**Head& neck**	18.6%	NA	22.9%	11.4%	8.9%
**Recurrence Rate, %** **(95% CI)**					
**MMS (%)**	1.5%	6.6%	0.0 % (0.0-16.8)	0.0 % (0.0-8.6)	5.5%
**WLE (%)**	7.4%	13.2%	3.6% (0.1-18.3)	13.2 % (4.4-28.1)	8.1%
**Recurrence rate in respective location**					
**Trunk & Extremities n (%)**	MMS1 (1.8%) WLE25 (17%)	NA	NA	MMS NA WLE 9.68%	Trunk 45% Extremities 33%
**Head& neck n (%)**	MMS 1(10%) WLE11 (47.8%)	NA	NA	MMS NA WLE 28.57%	22%
**Follow-up duration**	5.7 years	5 years (3-5.4)	5.8 years	5.4 years (2-15)	4.7 years
**Clinical question**	To compare long-term outcomes after MMS and WLE.	Efficacy of Mohs micrographic surgery	Wide excision or Mohs micrographic surgery for the treatment of DFSP	DFSP: wide local excision vs. Mohs micrographic surgery	To evaluate the cure rates of Mohs Tübingen technique (MTT) and wide local excision

**MMS:** Mohs micrographic surgery; **NA:** not available; **WLE:** wide local excision

**Table 2:** Summary of the characteristics of our meta-analysis

Summary Table: characteristic features of meta-analysis	Value*
Average Age of diagnosis	44.6 years
Female: Male Ratio	1.58
Average follow-up time for both groups	5.32 years
**Site of lesion**	
Trunk	52.80%
Extremities	31.75%
Head & Neck	15.45%
**Recurrence Rate**	
WLE	9.10%
MMS	2.72%
**Recurrence Rate by Region**	
**WLE:**	
Trunk & Extremities	13.34%
Head & Neck	38.19%
**MMS:**	
Trunk & Extremities	23.4%
Head & Neck	16.0%

**Table 3:** Forest plot showing OR with 95% CI of MMS and WLE comparing recurrence rates of DFSP by these surgical methods in comparative NRCT, Z=3.85(P=0.0001)

	MMS		WLE			Odds Ratio
Study	Events	Total	Events	Total	Weight	M-H,Random.95%CI
**DuBay D et al.**	1	71	6	87	7.7%	0.29[0.02, 1.64]
**Foroozan et al.**	10	90	53	174	65.8%	0.29[0.14, 0.59]
**Meguerditchian et al.**	0	20	1	28	3.3%	0.45[0.02, 11.55]
**Paradisi et al.**	0	44	5	38	4.1%	0.07[0.00, 1.28]
**Veronese et al.**	4	73	5	62	19.1%	0.66[0.17, 2.58]
Total (95%CI)		298		389	100.0%	0.31[0.17, 0.56]
**Total Events**	15		70			

Heterogeneity: Tau^2^=0.00; Chi^2^=2.54, df=4 (P=0.64); I^2^=0%

Test for overall effect: Z=3.85(P=0.0001)

**Table 4:** Quality assessment of included studies

	Risk of bias			Applicability Concerns			
Study	Patient Selection	Index Test	Reference Standard	Flow and Timing	Patient Selection	Index Test	Standard Reference
**DuBay *D et al.***	L	U	L	L	L	L	L
**Foroozan *et al.***	L	U	L	L	L	L	L
**Meguerditchian *et al*.**	L	U	L	U	U	L	L
**Paradisi *et al*.**	L	U	L	L	L	L	L
**Veronese *et al*.**	L	U	L	L	L	L	L

**L:** reference to low risk**; U:** reference to unclear risk**; H:** reference to high risk

## Current status of knowledge

Dermatofibrosarcoma protuberans (DFSP) which is a rare cutaneous soft tumor, is usually described as a slow growing with a very low metastatic potential [60] but has significant subclinical extension and great capacity for local destruction [9]. Historically, DFSP was first described in the 1890s by Taylor RW. and in the year 1924, Darier and Ferrand were credited for establishing DFSP as clinical pathological entity of a ‛progressive and recurring dermatofibroma [61,62], and a year later Hoffman,1925 established the term dermatofibrosarcoma protuberans [61,63]. The recommended mode of treatment for DFSP has been agreed to be surgery and is said to be curative but the latest concern was on the type of surgery to be done on this locally aggressive and highly recurrent cutaneous malignant tumor [64-66]. The decision to choose the type of surgery to use is based on several factors which include but not limited to the following: how capable and advanced the institution is to hand meticulous complex operations, clinicians experience, location of the lesion, the presentation of the lesion; early or late presentation. However, size and anatomic site of tumor has being recognized as one of the most important factors influencing the choice of surgical modality (WLE vs MMS) and adjuvant radiation or chemotherapy for most clinicians [66]. Thus, our meta-analysis was conducted in order to assist and guide clinicians on the favorable surgical method in view of the different clinical scenarios the patients can present with. The meta-analysis reviewed that Mohs Microscopic Surgery (MMS) is superior to Wide Local Excision in terms of reduction on the rate of recurrence of the tumor. The pooled adjusted odds ratio (OR) analysis, indicated that there was a direct relationship with regards the reduced recurrence rate of DFSP in the MMS group compared to the WLE group (OR: 0.31;95% CI :0.17-0.56) as shown in Table 3 [57-59]. Furthermore, there was significant association between the reduced recurrence rate with the MMS in DFSP patients with a statistical P-value of 0.0001 at 95% CI. This could be extrapolated to indicated that the cure rate is DFSP is directly proportional to reduced recurrence rates. Thus, the MMS group has a fairly high cure rate than the WLE. It should be recommended as the first treatment option but in less advanced centers WLE surgery could be done but with a 3 cm free margin consideration (2-5cm) [52,67].

The study included 5 retrospective reviews, trials and studies of non-randomized controls trials (NRCT). Quality assessment of the studies eligible in the meta-analysis is as indicated in Table 4 [57-59]. They were fairly representation although Asia and Africa were not represented. Literature Kreicher KL *et al.* documents that the incidence of DFSP is higher in the black population [68] than white at a rate of almost 2 times(95% CI of rate ratio: 1.8-2.1 )[69]. The countries that comprised our study was United states of America (U.S.A), Italy and the United kingdom (U.K). Most of the studies was on a multicenter review partly due to the rarity of the condition and the search span was for over 10 years for most of the institutions. Our study had a pooled patient load of 684 for both WLE and MMS group with the smallest study group done in the UK, Canada by Meguerditchian *et al.* with a sample size of 48 [57]. Their study showed that MMS is far superior to WLE in terms of local recurrence rate which was recorded at 0% in comparison to 3.6% at a median follow-up of 40.4 months for MMS and 49.9 months for WLE respectively. This study was categorized as level B in terms of quality of evidence according to criteria by Ebell *et al.* [70], and Robinson *et al.* [71].The study had a larger percentage of patients in the MMS category with lesions on the trunk and extremities at 39.6% and 37.5% respectively. The average age of diagnosis for our meta-analysis was at 44.6 years and had more females than males at 1.58. All studies showed and increased predominance of females the highest being at 2.24 by DuBay *et al.* and lowest at 1.18 by Veronese *et al.* and Meguerditchian *et al.* These findings agree with most of the current literature on incidence of DFSP [68,69,72]. The average follow-up for patients in meta-analysis was 5.32 years and some studies showed much earlier rates of recurrence like for Paradisi *et al.* which was under 2 years. A patient with primary and later secondary DFSP on his right shoulder, underwent five consecutive local recurrences at 1, 2, 4, 6 and 7 years respectively from the first date of excision with the traditional wide technique (referring to the WLE). The reason for recurrence was not documented neither was the histological variant of the DFSP tumor [58].The possible recurrence in this patient, could be failure to completely resect the tumor due to care of not damaging other surrounding structures and maybe lack of experience in flap or closure techniques thus tried to maintain cosmesis. The meta-review did not categorize the lesions as either being primary or secondary in the various parts. The trunk is the commonest site for the DFSP lesion which was at 52.80% for the pooled value with the extremities (both upper and lower) at 31.75% and combined head and neck regions being the lowest site of lesion at 15.45%. There is no attributable reason as to why the lesion has a predilection to the trunk. It calls for further investigation and evaluation.

Traditionally, it had be accepted that deep and wide local excision (WLE) had been considered the Gold Standard treatment of this locally invasive tumor [73-75]. WLE in the management of DFSP is defined as a surgical procedure to remove a small area of diseased or problematic tissue with a normal margin of tissue of above 2 to 5cm [63,76-78]. Malumani *et al.* recommends that a 3 cm free margin zone is recommendable in WLE. It has better outcomes in relation to cure rates but other confiding factors should be considered in every case. Factors like the size and site of tumor, the ease of tumor margin identification, the type of flap to be used and reconstructions techniques et cetra [65]. The recurrence rate of DFSP in our meta-analysis at was at 9.10% and 2.72% for the WLE and MMS groups respectively. Ideally, in the MMS group we would not expect any recurrence because this surgical procedure involves the microscopically controlled surgery used to treat most common types of skin tumors like DFSP, Melanoma et cetra. During the surgical procedure, after each removal of the lesion and while the patient waits, the tissue specimen is examined microscopically for tumor cells. That examination aides the surgeon decides if further tissue removal is necessary. Mohs surgery is one of the few surgical methods of obtaining complete margin control during skin cancer removal (a complete circumferential peripheral and deep margin assessment) using frozen section histology technique [79,80]. Minton, 2008. Decribes MMS as a tissue-sparing technique that allows for excision of tumor under complete microscopic control and thus boasts of very narrow surgical margin and high cure rates [81].

The 2.72% recurrence rate in the MMS group is quiet high for a technique that consumes a lot of time and guarantee is given before repair and closure of the surgical wound that the margins are free of any tumor cells. These observations bergs questions of histopathological error or another de-novo recurrence of DFSP in the scar region or seeding of the tumor cells during the procedure. Some studies included in this meta-analysis had zero percent recurrence rate like Meguerditchian *et al.* [57] and Paradisi *et al.* [58]. Foroozan *et al.* had the highest rate of recurrence rate of 6.6% in the MMS category. This high value could be the cause of the slightly high rate of 2.72% in our meta-analysis. The author notes that in the Foroozan *et al.* study, one patient had tumor recurrence within fourteen months after the MMS procedure, although data on tumor and patient characteristics were not available for this recurrence. However, the quality of included studies was level B grade according to criteria by Robinson *et al.* [71]. The study also labored to analyze the recurrence rate in view of the site of lesion. The body regions were grouped in two zones in view of the increased chances of complications both from the resection of tumor and degree of difficulty and complexity in the flap repair and reconstructive surgery. The zones are: the head and neck zones and the second involved the trunk and the extremities. The recurrence rate by regions was highest in the WLE head and neck zone at 38.19%. This was expected in view of the surgical procedure done where the operator considers the anesthetic consequences of a large free margin cut, also in anticipation of possible complications. Terro *et al.* described a case report of a Syrian refugee who had a DFSP tumor on his left cheek, the 10 × 8 × 4 cm^3^-sized tumors were entirely removed via en bloc wide local excision with clear margins of about 3 cm. The excision included some branches of the facial nerve. In view of the resultant large defect and inability to adequately rotate local tissue, thus a lower trapezius musculocutaneous pedicle flap from the back was deployed and there was no evidence of recurrence after 32 months of follow-up [2]. Other authors have described cases in the head and neck zones [7,8,82].

The trunk and extremities zone in the WLE were at 13.34%. This is slightly half that in the head and neck zone. The picture is quiet the reverse in the MMS group. There are more recurrences in the Trunk and extremities zone of 23.4% as compared to 16.0% in the head and neck zone. The author advocates the employment of MMS, it has advantages in controlling the tumor burden microscopically, but it is laborious, technically demanding, expensive, and time-consuming, which are major disadvantages [64,83]. Many authors suggested the importance of deep margin control while describing clinical cases with suspected deep tissue invasion [32,84,85]. Particularly in recurrent lesions, the author recommends the use of MMS or any appropriate Modified MMS [59,85]. Post-surgical radiation can also be used as a treatment option in patients who might have involved margins or lesions that are unresectable. This can be applicable to the Head and neck zones where resection can be a bit of a challenge. Haas *et al.* [86] in a case series of 38 patients demonstrated a local control probability of 82% with post-operative radiation compared to 67% after surgery alone. Ballo *et al.* also demonstrated a 95% control rate with adjuvant radiotherapy in 19 patients, with a 6-year follow-up [87]. The first effective neoadjuvant systemic chemotherapy in the management of DFSP was imatinib, which demonstrated high anti-tumor activity in advanced cases [27,74]. Rutkowski *et al.* described results of their study which indicated the long-term activity of imatinib in therapy of inoperable and metastatic cases of DFSP, including fibrosarcomatous variant. Also, some DFSP patients were evaluated as having unresectable tumors initially and with potential metastatic lesions or necessitating a mutilating type of surgery, turned out resectable after a course of imatinib therapy and this rational approach leading to complete remission maybe potentially curative [74,88-91]. Since even in the MMS group there are cases of recurrence of DFSP, this observation bergs a further study of comparing MMS only verses MMS with appropriate adjuvant therapy and MMS only versus WLE with appropriate adjuvant therapy.

## Conclusion

Our study reviewed that MMS has better outcomes that WLE in terms of postulated cure rates and the head and neck zone are associated with higher chances of recurrence rate despite the clinical variant of DFSP. It has also been observed that neoadjuvant chemotherapy with imatinib as a pre-surgical course in therapy of inoperable and/or metastatic cases of DFSP, including fibrosarcomatous variant has turned these debilitating tumors into resectable with remission and potentially curative. On the other hand, radiation therapy is recommended as a post-surgical procedure if there is suspicion of positive margins. Thus, in high recurrence zones an augmented approach is recommended.

### What is known about this topic

- Dermatofibrosarcoma protuberans (DFSP) which is a rare cutaneous sarcoma, is a locally aggressive, and low metastatic potential with high recurrence rate;
- Wide local Excision has been the gold standard of treatment although Mohs Micrographic Surgery is being advocated as preferred choice;
- Surgeons have recommended a 2-5 cm clear margin in the surgical management of DFSP.

### What this study adds

- Our study affirms that Mohs Microscopic Surgery is superior to the traditional Wide Local excision in view of reduced recurrence rates hence increased cure rate despite its limitations of longer operation time and requires a Multidisplinary team in one setting;
- In the advent of late presentation or tumor in high recurrence zones like the head and neck regions, pre-surgical chemotherapy with Imatinib and Post-surgical radiotherapy has shown better outcomes;
- In resource challenged centers, WLE can be used with a 3-5 cm clear margin cut, with or without pre or post-surgical therapy and a longer duration of follow-up, over 5 year has been associated with decreased morbidity and mortality.

## Competing interests

The authors declare no competing interests.
